# Deep-Learning-Based Power Generation Forecasting of Thermal Energy Conversion

**DOI:** 10.3390/e22101161

**Published:** 2020-10-15

**Authors:** Yu-Sin Lu, Kai-Yuan Lai

**Affiliations:** Green Energy and Environment Research Laboratories, Industrial Research Institute (ITRI), Hsinchu 310, Taiwan; yslu@itri.org.tw

**Keywords:** deep learning, recurrent neural network, power generation prediction

## Abstract

ORC is a heat to power solution to convert low-grade thermal energy into electricity with relative low cost and adequate efficiency. The working of ORC relies on the liquid–vapor phase changes of certain organic fluid under different temperature and pressure. ORC is a well-established technology utilized in industry to recover industrial waste heat to electricity. However, the frequently varied temperature, pressure, and flow may raise difficulty to maintain a steady power generation from ORC. It is important to develop an effective prediction methodology for power generation in a stable grid system. This study proposes a methodology based on deep learning neural network to the predict power generation from ORC by 12 h in advance. The deep learning neural network is derived from long short-term memory network (LSTM), a type of recurrent neural network (RNN). A case study was conducted through analysis of ORC data from steel company. Different time series methodology including ARIMA and MLP were compared with LSTM in this study and shows the error rate decreased by 24% from LSTM. The proposed methodology can be used to effectively optimize the system warning threshold configuration for the early detection of abnormalities in power generators and a novel approach for early diagnosis in conventional industries.

## 1. Introduction

In today’s society, more than 90% of energy is generated as thermal energy, most of which is emitted into the environment as waste heat after use. Only 40–50% of thermal energy is converted to manufacturing thermal energy, mechanical power, electricity, or chemical energy. In particular, low-temperature waste heat (less than 100 °C) constitutes 63% of global waste heat [1]. Waste heat from thermal energy applications has led to problems such as energy waste, thermal pollution, and global warming. For example, when waste heat is discharged into lakes and rivers, the water temperature will rise sharply, causing the death of animals and plants. It may also increase the heat in the atmosphere and affect global climate change. The recovery and reuse of waste heat is an effective way to reduce thermal pollution. Recycling schemes for low-temperature waste heat are highly conducive to energy conservation and carbon emission reduction. Such schemes also help governments reduce the load on electrical grids. Therefore, countries worldwide have invested in research and development as well as commercialization of lower-temperature thermal energy conversion technology in the past decade. In particular, organic rankine cycle (ORC) products are advantageous for their use of mature technology as well as high reliability and low costs. Such products are considered the most economical solution to low-temperature thermal energy conversion with the highest conversion efficiency [2]. At present, ORC technology is widely used in industrial waste heat recycling [3] and geothermal energy conversion [4]. In the case of industrial waste heat, which is emitted continuously for 24 h, recycling and applying it to power generation can achieve a capacity factor of >75%, and the generated power can contribute to the baseload. The capacity factor stated here shows the operation time of power generation system from waste heat recovery can last for a year, about 6500 h. The operation time excludes the factors of unit maintenance and factory maintenance. Accordingly, the effective utilization of low-temperature waste heat creates multiple benefits in the areas of energy conservation, carbon emission reduction, environmental protection, and economic development.

In recent years, deep learning technology has been used to perform time series forecasting in the energy field. In solar energy applications, autoencoders and long short-term memory (LSTM) deep learning frameworks are used to estimate solar cell power conversion, and the forecasting results are more accurate compared with those of physical models [5,6,7]. Deep learning is also applied to photovoltaic power forecasting [8]. In addition, deep learning has been used to predict wind speed and wind power generation, achieving higher accuracy compared with conventional time series analysis [9,10].

Currently, ORC applications for estimating power generation from thermal energy conversion cannot effectively predict the amount of power generated in real time. Solutions are devised only after problems in power generators are generated. More clearly, we cannot effectively know the generator failure early, and we must wait for the abnormality before inspecting and repairing it. The advancement of computer hardware has considerably enhanced the computation ability of computers. This drives the rapid development of deep learning technology, which is now widely adopted in research, and allows for time series analysis with more detailed data structures. In deep learning, scholars have achieved major breakthroughs in the practical applications of recurrent neural networks (recurrent neural networks, RNNs), which are now widely used in article generation, machine translation, voice recognition, and time series analysis. From the literature review related to applying established deep learning to wind, solar, and photovoltaic power forecasts, the deep learning methods are capable of making forecast analysis on thermoelectric power generation.

In this study, we proposed a deep learning framework. The amount of power generated, working fluid volume, cold and heat source temperatures, and pressure during the operation of ORC systems were used as training data for an RNN. The actual amount of power generated was used to verify and test the forecasting results of the RNN to develop a network model that can forecast power generation for the next 12 h. The proposed model can be used to monitor the condition of power generators and effectively configure warning thresholds to detect system abnormalities 12 h before they occur. This allows maintenance personnel to schedule system diagnosis and repair in advance.

Currently, most thermal energy conversion units are maintained on a regular basis. This leads to problems such as the unnecessary replacement of healthy parts and downtime caused by temporary faults, which result in the waste of resources and reduced utilization rates. To effectively increase the amount of power generated, power companies mostly adopt Internet of Things schemes as a solution. In general, sensors are installed on essential parts to collect data, which are transmitted to a cloud center through online communication. The real-time monitoring platform in the cloud center allows companies to monitor their generators remotely. Additionally, warning thresholds can be configured in such platforms. When abnormalities are detected, notifications are immediately sent to the personnel in charge for subsequent maintenance [11]. Using our proposed method, it is expected to be able to predict abnormalities in ORC.

**Figure 1:**
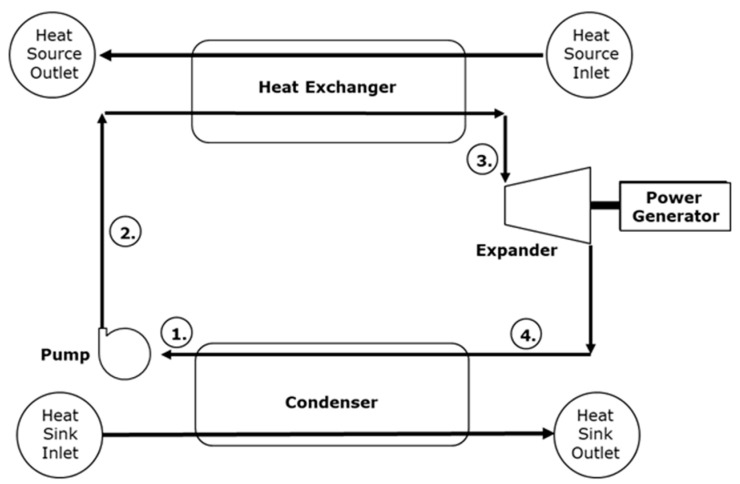
Key components in an ORC system.

The ORC was proposed by Tabor and Bronicki [12]. An ORC system uses an organic fluid as a medium to absorb thermal energy and generate power. Figure 1 illustrates the configuration of the ORC, which is divided into four components. The key components and principles of the ORC are described as follows: (1) Pressure pump: a pump is used to increase the pressure of a low-pressure working fluid and transport the liquid to the heat extractor. (2) Heat exchanger: an evaporator is used to extract thermal energy from the heat source and heat the working fluid in the loop system. This converts the working fluid to saturated gas or overheated gas, which is then directed to the working component. (3) Thermal power converter: The working components of an ORC system include a turbine expander, screw expander, or scroll expander. The working component module guides the gaseous working fluid into the expander, which converts the pressure and temperature of the working fluid into shaft power that enables a generator to generate power. (4) Heat removal component: finally, a condenser is used to remove the excess thermal energy from the gaseous working fluid and condense the fluid to liquid form. This completes one thermal cycle in the ORC system. In general, a heat source and a cold source with a temperature difference of ≥10 °C are applicable to ORC systems, and such systems become profitable when the temperature difference is ≥50 °C. Suitable working fluids, heat exchangers, and heat sinks can be employed in an ORC system along with diverse cold and heat sources to generate power. The temperature difference between thermal energy in room and cold temperatures may also be used to generate power through ORC systems.

Our work focuses on combining ORC mechanical heat flow knowledge with data science. Using deep learning, time series analysis for ORC operating data, we hope to effectively predict ORC power generation. We propose improvements based on the concept of LSTM model and compare some commonly used time series models. According to the experimental results, our improved model can have the best prediction performance in the case of predicting the next 12 h.

## 2. Methods

### 2.1. Recurrent Neural Networks

A common type of neural network is the feedforward neural network, in which data are transmitted through multiple layers in one direction from the input to output layers. This means that all layers are independent of each other. However, neighboring data points in a time series data set are in a sequential relationship, in which the current input and subsequent input are temporally correlated. Therefore, the time series input was used to train the prediction model. RNNs differ from feedforward neural networks in that they can process sequential data as well as memorize the recent output of a hidden layer node and use it as the extra output in the next hidden layer. This enables RNNs to account for the temporal relationships between data points [13].

**Figure 2:**
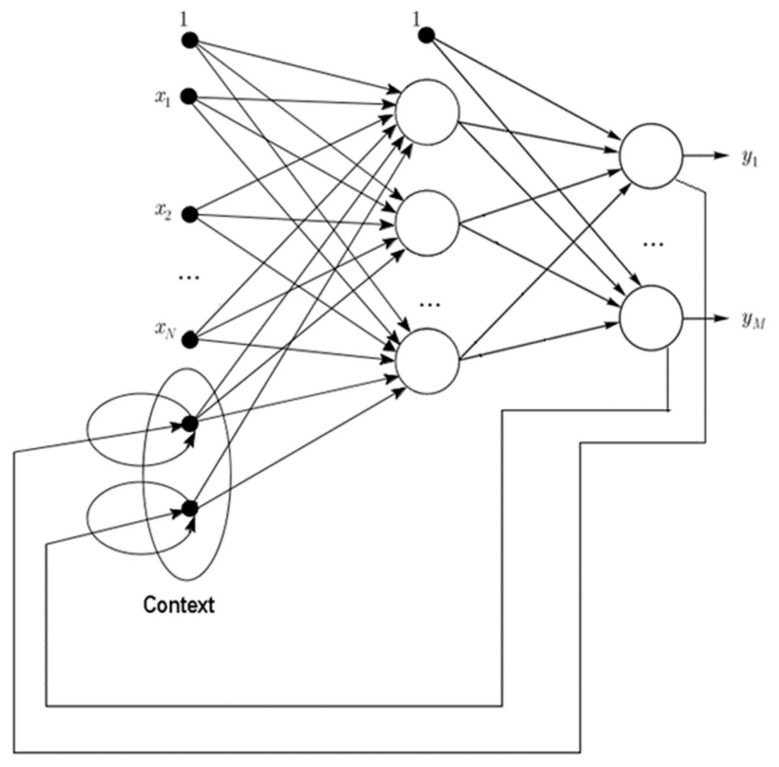
Framework of a Jordan network.

**Figure 3:**
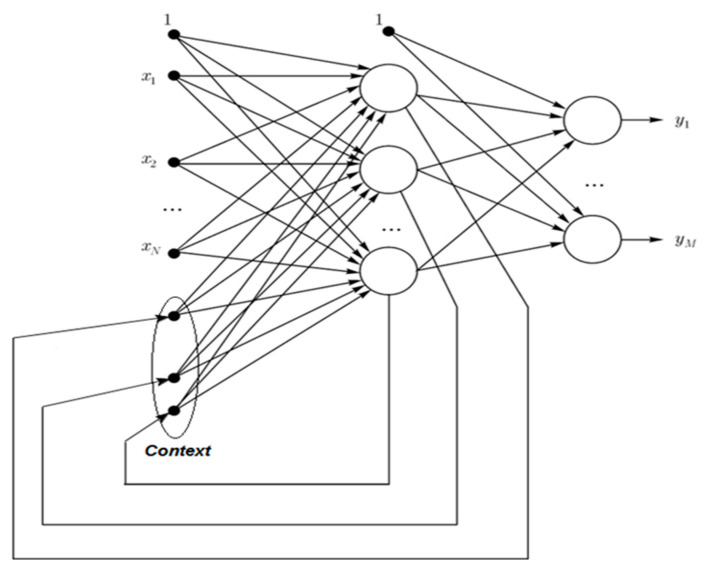
Framework of an Elman network.

Tenti [14] mentioned numerous types of RNN with different frameworks, such as those shown in Figure 2 and Figure 3 and Equations (1) and (2). The equations indicate that in a Jordan network, the output ($y_{t-1}$) of the previous output layer is memorized and used as the other output of the current hidden layer. By contrast, an Elman network memorizes the previous hidden layer output ($h_{t-1}$) and uses it as another output of the current hidden layer. Elman networks are more widely used in applications, including famous artificial neural network open source platforms, such as TensorFlow and Keras.

(1) $$h_{t}{=\sigma }_{h}\left(W_{h}x_{t}{+R}_{h}y_{t-1}{+b}_{h}\right),\;y_{t}{=\sigma }_{y}\left(W_{y}h_{t}{+b}_{y}\right),$$

where $h_{t}$ is the current (*t*) hidden layer output, $y_{t}$ is the current (*t*) output layer output, $x_{t}$ is the current (*t*) output vector, σ is the activation function, *W* and *R* are the corresponding weights, *b* is the corresponding bias, and $y_{t-1}$ is the previous (*t* − 1) output layer output.

(2) $$h_{t}{=\sigma }_{h}\left(W_{h}x_{t}{+R}_{h}h_{t-1}{+b}_{h}\right),\;y_{t}{=\sigma }_{y}\left(W_{y}h_{t}{+b}_{y}\right),$$

where $h_{t}$ is the current (*t*) hidden layer output, $y_{t}$ is the current (*t*) output layer output, $x_{t}$ is the current (*t*) output vector, σ is the activation function, *W* and *R* are the corresponding weights, *b* is the corresponding bias, and $h_{t-1}$ is the previous (*t* − 1) hidden layer output.

### 2.2. Long Short-Term Memory Networks

Proposed by Hochreiter and Schmidhuber [15] in 1997, LSTM networks are a derivative of RNNs. An Elman network maintains short-term memories through recurrent connection with outputs in neighboring hidden layers. However, because long-term dependencies are required for time series analysis, LSTM networks, a new type of RNN, were developed to maintain long-term memories through gradual and slow changes in weights.

**Figure 4:**
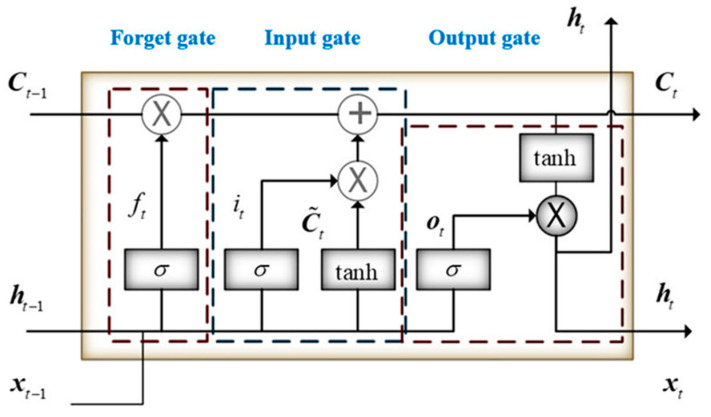
Framework of an LSTM network.

Long-term memories have great potentials in applications such as sentence analysis and voice processing. The unique model structure of an LSTM network overcomes problems regarding the vanishing and exploding of error signals during backpropagation. Therefore, such networks are applicable to the processing of time series data with long intervals. Figure 4 illustrates the internal framework of an LSTM network.

In Figure 4, *σ* is the S function, *x* is the output, $h_{t-1}$ is the previous hidden layer state, *W* is the corresponding weight of the input, *R* is the iteration weight, and *b* is the bias. The initial cell state and hidden state are 0.

The forget gate ($f_{t}$) determines the extent to which a cell forgets its old state ($C_{t-1}$).

(3) $$f_{t}=\sigma \left(W_{f}x_{t}{+R}_{f}h_{t-1}{+b}_{f}\right).$$

The input gate ($i_{t}$) determines the extent to which a new input updates a cell state ($C_{t}$).

(4) $$i_{t}=\sigma \left(W_{i}x_{t}{+R}_{i}h_{t-1}{+b}_{i}\right).$$

A cell state ($C_{t}$) denotes a long-term memory, which is divided into two parts: (1) The new input is subject to the tanh activation function and enters a temporary state (${\bar{C}}_{t}$). (2) The forget gate allows the cell to remove unnecessary data, and the input gate inputs new data to update the cell.

(5) $${\bar{C}}_{t}=tanh\left(W_{\bar{c}}x_{t}+R_{\bar{c}}h_{t-1}+b_{\bar{c}}\right).$$

(6) $$C_{t}=f_{t}{*C}_{t-1}+i_{t}{*\bar{C}}_{t}.$$

The output gate ($O_{t}$) determines whether the new cell state ($C_{t}$) can be adopted as the new hidden state.

(7) $$O_{t}=\sigma \left(W_{o}x_{t}{+R}_{o}h_{t-1}{+b}_{o}\right).$$

A hidden state ($h_{t}$) denotes a short-term memory that serves as the input of the next hidden layer.

(8) $$h_{t}{=O}_{t}{*tanh(C}_{t}).$$

## 3. Experimental Design and Simulation

### 3.1. Data Set and Data Pre-Processing

**Figure 5:**
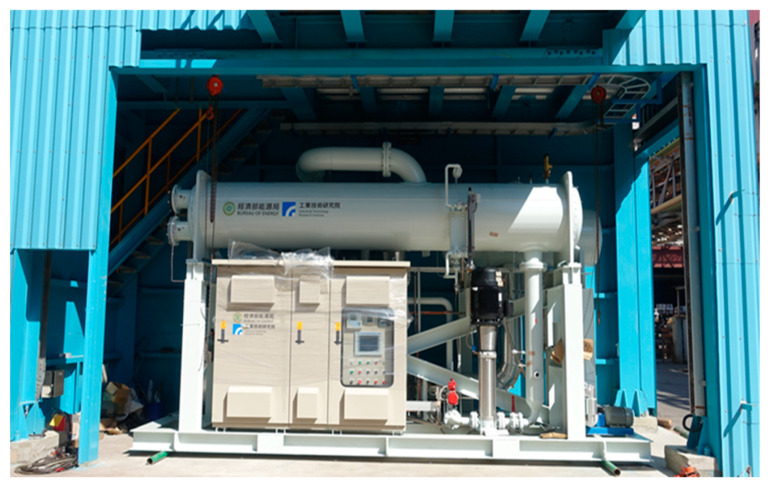
A photograph of the 200-kW organic Rankine cycle system. Source: Industrial Technology Research Institute (ITRI).

The data analyzed in this study came from a waste heat recycling generator used in steel manufacturing. The research site employed the 200 kW ORC generator developed by the present research team (Figure 5). The original data set incorporated more than two million pieces of data from October 2016 to May 2020. Each piece of data incorporated 41 parameters, the more essential of which were net power generation, working fluid volume, heat source temperature and pressure, and cold source temperature and pressure. To predict power generation for the next 12 h, the original data were averaged by the hour. In order to raise the accuracy through more data, the study made the model learns by every 15 min or 1 min. However, 1 to 15 min prediction in advance is way too short for maintenance staff to deal with the situation. For the multi-step deep learning model, the longer the sequence is, the less accurate the prediction model is. To predict the next 12 h generation, it must be made by 48 steps or 720 steps in advance respectively and the accuracy will be less than the 1-h data. The correctness of the data was verified by removing erroneous data caused by damaged parts. Accordingly, 18,000 pieces of data were retained for analysis. Table 1 lists the number of data items, maximum and minimum values, mean, and standard deviation of the net power generation after pretreatment.

**Figure 6:**
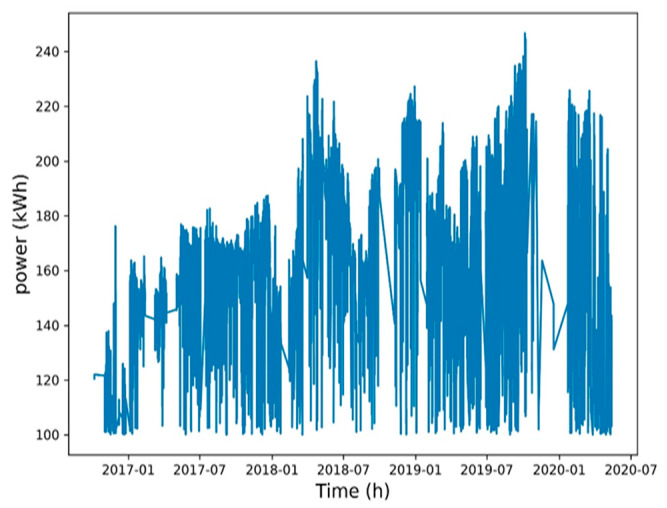
Net power generation.

**Figure 7:**
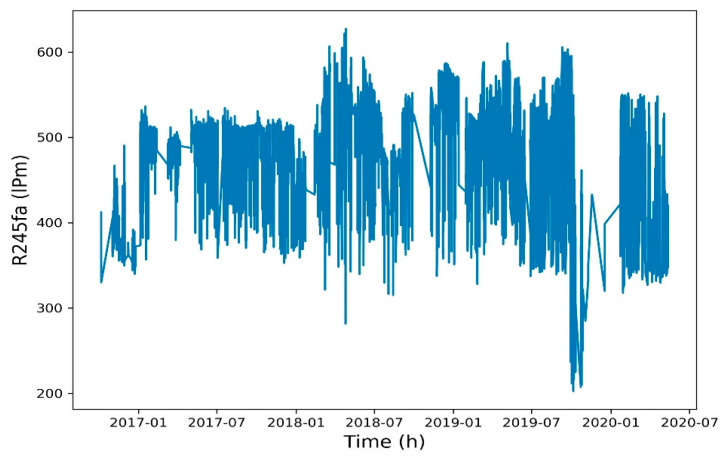
Working fluid volume.

**Figure 8:**
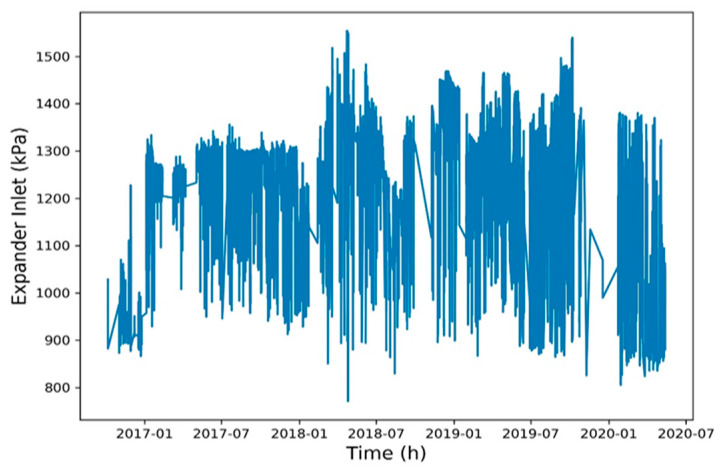
Expander inlet pressure.

**Figure 9:**
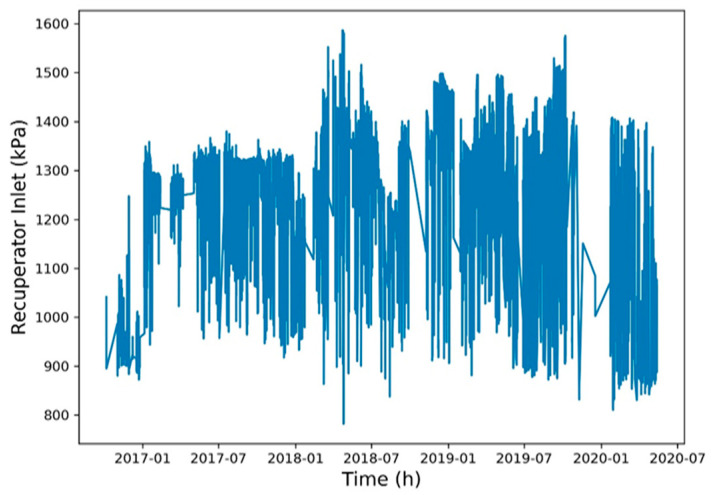
Recuperator inlet pressure.

**Figure 10:**
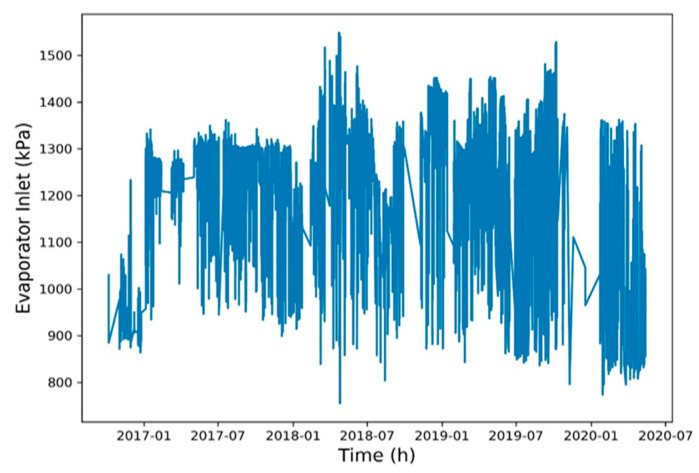
Evaporator inlet pressure.

**Figure 11:**
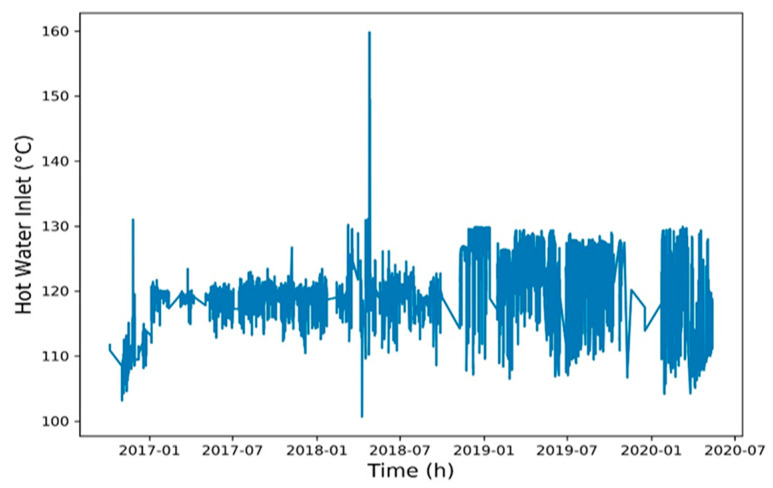
Heat source inlet temperature.

**Figure 12:**
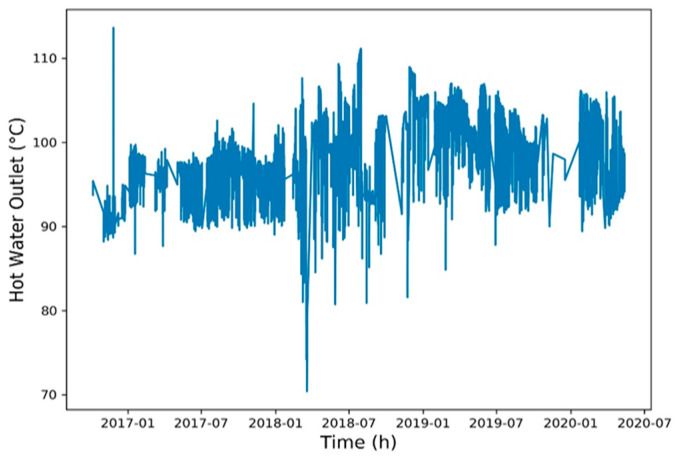
Heat source outlet temperature.

To reduce the training time, a Spearman correlation matrix was used to select model training parameters with a correlation coefficient of >0.7 or <−0.7. The selected parameters were working fluid volume, net power generation, expander inlet pressure, recuperator inlet pressure, evaporator inlet pressure, and heat source outlet temperature (Figure 6, Figure 7, Figure 8, Figure 9, Figure 10, Figure 11 and Figure 12). The *x*-axis of the chart represents time, and the *y*-axis represents the corresponding unit. The chart can effectively show the trend of pre-processing parameters used to predict power generation. The correlation coefficient is used to reflect the close relationship between variables and find out the parameters highly related to power generation.

### 3.2. Model Parameters

In the pretreatment, 14,400 pieces of data were used as the training set, and the remaining 3515 pieces of data were used as the testing set. The mean and standard deviation of the training set were used to normalize all data. The normalization equation was as follows: (original value−mean)/standard deviation. The training set and test set are divided into eight to two, and the standard method is standard deviation normalization. This usage is a normal operation in data science.

In Equation (9), n is the total number of data, $y_{i}$ is the normalize value, and $x_{i}$ is the original value.

(9) $$y_{i}=\frac{x_{i}-\bar{x}}{s},\;\bar{x}=\frac{1}{n}\overset{n}{\underset{i=1}{\sum }}x_{i},\;s=\sqrt{\frac{1}{n-1}\overset{n}{\underset{i=1}{\sum }}{\left(x-\bar{x}\right)}^{2}}$$

**Figure 13:**
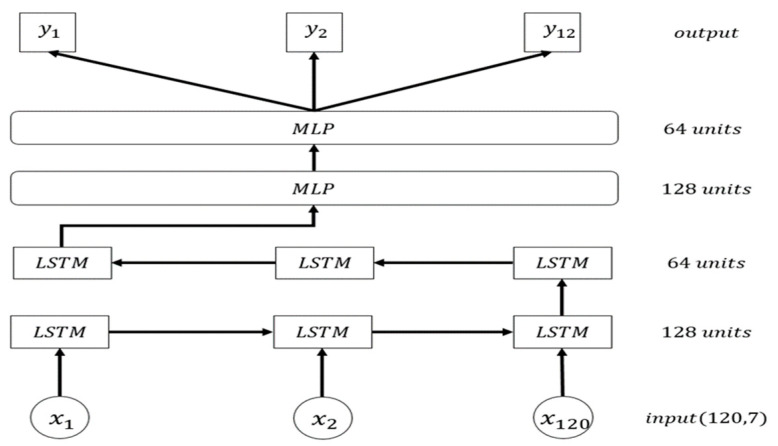
Multivariate LSTM model for predicting the amount of power generated in the next 12 h.

Univariate and multivariate LSTM models were proposed in this study. Historical data must be used to predict future power generation. In the univariate model, 120 h of data were used to predict the power generation in the next hour. The hidden layer comprised a double-layered LSTM model with 128 neurons in one layer and 64 neurons in the other. They were connected to two connection layers that had 128 and 64 neurons, respectively. The output layer had one neuron to predict the amount of power generated for the next hour. The multivariate model used 120 h of data from the past 5 days to predict the amount of power generated for the next 12 h. The design of the hidden layer was identical to that of the univariate model. The output layers consisted of 12 neurons to predict the amount of power generated for the next 12 h (Figure 13).

### 3.3. Model Accuracy Assessment

To effectively assess the accuracy of the model in predicting power generation, three assessment methods were adopted, namely the mean absolute error (MAE), root mean square error (RMSE), and mean absolute percentage error (MAPE) [16]. The three methods differ in mathematical significance and exhibit distinct advantages and disadvantages; hence, they were adopted together to perform a comprehensive comparison.

In Equation (10), n is the total number of data, y is the observed value, and $\hat{y}$ is the predicted value.

(10) $$\mathrm{MAE}=\frac{\sum_{i=1}^{n}|{\hat{y}}_{i}-y_{i}|}{n}$$

The MAE can be used to reflect prediction errors and is in the range of (0, +∞). When the predicted and observed values are identical, the MAE is 0, indicating a perfect model. Large errors lead to large MAE values.

(11) $$\mathrm{RMSE}=\sqrt{\frac{\sum_{i=1}^{n}{\left({\hat{y}}_{i}-y_{i}\right)}^{2}}{n}}$$

The RMSE is used to observe the difference between the observed and predicted values. However, the difference between the values might be difficult to observe following normalization. The RMSE is also in the range of (0, +∞). When the predicted and observed values are identical, the RMSE is 0, indicating a perfect model. Large errors lead to large RMSE values.

(12) $$\mathrm{MAPE}=\frac{100\%}{n}\sum_{i=1}^{n}x_{i}|\frac{{\hat{y}}_{i}-y_{i}}{y_{i}}|$$

The MAPE is presented as percentage values. The error of each predicted value is divided by the observed value; hence, skewness can occur. This means that the MAPE is considerably affected when an observed value is extremely low and the error is large. Consequently, optimization based on the MAPE can lead to illogical prediction results. The MAPE is in the range of (0, + ∞). When the predicted and observed values are identical, the MAPE is 0%, indicating a prefect model. Large errors lead to large MAPE values.

### 3.4. Autoregressive Integrated Moving Average and Multilayer Perceptron Models

This section introduces two time series analysis models for comparison with LSTM models.

Autoregressive integrated moving average (ARIMA) [17]: The ARIMA model is a time series analysis method based on statistical theories. ARIMA models built using collected data can be used to observe the trend, seasonal characteristics, and irregular patterns of the data. Such models are adjusted using the parameters *p*, *d*, and *q*; *p* and *q* are the order of the autoregressive model and moving average model, respectively, and d is the calculated difference.

Multiplayer perceptron (MLP) [18]: The MLP model is a type of feedforward neural network that produces a set of outputs from a set of inputs. MLP models are composed of multiple node layers that are connected to each other. Except for the input node, all nodes are neurons with nonlinear activation functions. Of the two hidden layers in the MLP model of this study, one had 128 neurons and the other had 64 neurons. The output layer had either 1 or 12 neurons depending on the number of prediction steps. This study’s MLP model can be considered an LSTM model without the LSTM structure. The accuracy of the MLP model was compared with that of the designed LSTM model.

In addition, this study compared the accuracy of the multivariate and univariate models. The univariate model only used the previous amount of power generated to make a prediction for the next 12 h. By contrast, the multivariate model accounted for the working fluid volume, net power generation, expander inlet pressure, recuperator inlet pressure, evaporate inlet pressure, heat source inlet temperature, and heat source outlet temperature.

## 4. Results and Discussion

**Figure 14:**
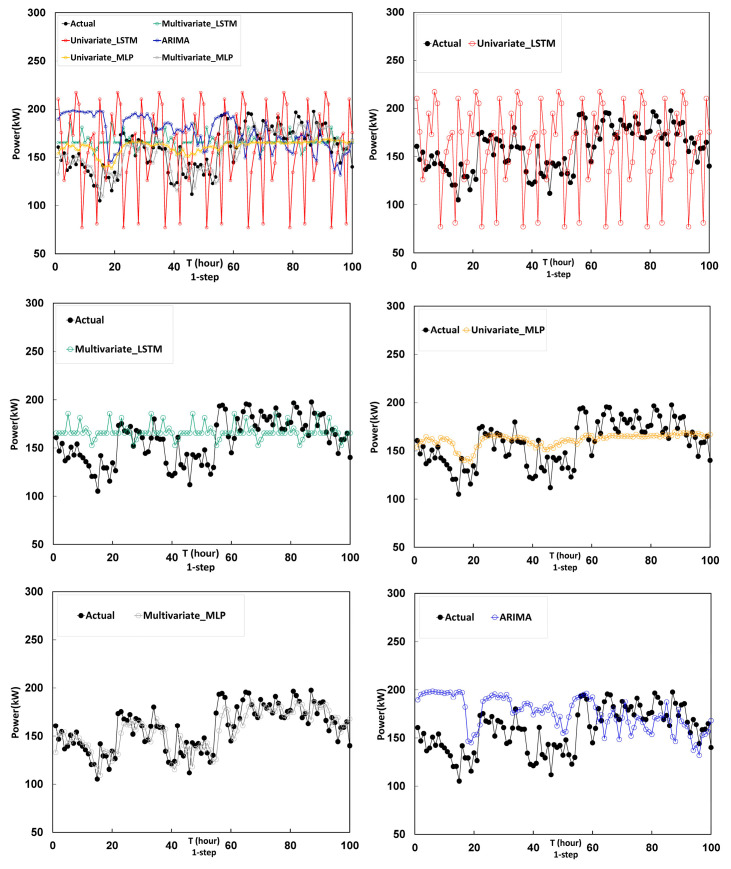
Prediction results of one-hour-ahead forecasting models by univariate LSTM, multivariate LSTM, univariate MLP, multivariate MLP, and ARIMA.

**Figure 15:**
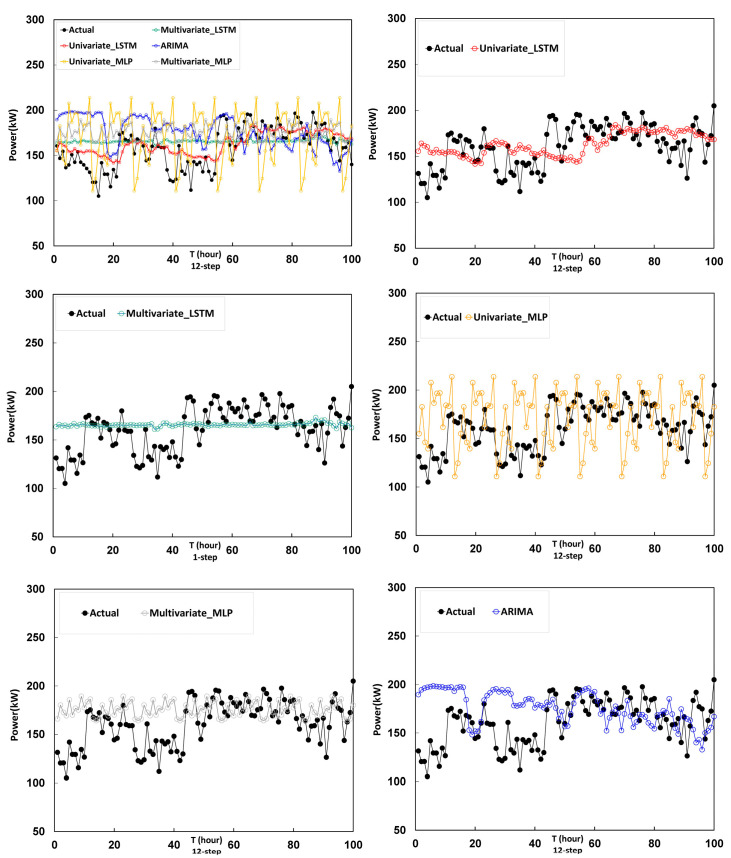
Prediction results of 12-h-ahead forecasting models by univariate LSTM, multivariate LSTM, univariate MLP, multivariate MLP and ARIMA.

To validate the prediction performance of the multivariate LSTM model for 1 h and 12 h, five models are employed for comparison: the Univariate LSTM model, the multivariate LSTM model, the Univariate MLP model, the multivariate MLP model and the ARIMA model. The evaluation indexes of the proposed and comparison models are presented in Table 2. The forecasting results based on the Univariate LSTM, multivariate LSTM, univariate MLP, multivariate MLP, and ARIMA models are shown in Figure 14 and Figure 15.

Based on the forecasting results of one-hour-ahead forecasting models shown in Figure 14, the following can be observed. The ARIMA model almost mapping to true data after 50 h and performance is best. The prediction result of the LSTM model is similar to the concept of mean value, and its performance is not bad. The MLP model is a backward indicator, and the previous hour’s data is used as the predicted value.

Based on the forecasting results of twelve hour-ahead forecasting models shown in Figure 15, the following can be observed. Results are about the same as predicted in the next hour. Under the fluctuation of the data, the LSTM model tends to the average of the data, which makes this method have a certain degree of stability for prediction work.

The results revealed that when making a prediction for the next hour, the ARIMA model was the most accurate, and using multiple variables reduced the model accuracy (Table 2). This indicated that the data set used in this study was from a stable generator. Therefore, using the mean amount of power generated alone achieved excellent model accuracy, whereas using multiple variables to perform the prediction resulted in excessive noise signals during model training. When the multivariate MLP was used as the benchmark, the prediction error of the LSTM models assessed using RMSEs were reduced by 27% compared with those of the MLP models.

When making a prediction for the next 12 h, the ARIMA model performed poorly, because of the large variability and uncertainty of the data in this case. The accuracy was reduced by 90% compared with other deep learning models. In addition, the multivariate models performed more favorably than did their univariate counterparts. When the univariate MLP model was used as the benchmark, the network prediction error of the LSTM models assessed using MAE was reduced by 24% compared with those of the MLP models. MLP only considers the weight of the current time point. LSTM uses the weight of the past time point as the prediction standard, so it is reasonable that LSTM predicts better than MLP.

In the case of predicting a single step, the accuracy performance of Multivariate MLP is less than Univariate MLP due to the over-fitting which occurs in training process from the growing accuracy of parameters. It happens when the result of prediction model is similar to the training data and cause poor accuracy of the test set. However, this phenomenon does not occur in multiple steps. The multi-step forecasting is relatively difficult to accurately simulate the data, so there will be no overfitting problems.

Regarding the limitations of the model, the neural network-like method belongs to supervised learning, and the prediction model must be learned based on past historical data. If the historical data does not represent this model or the historical data contains too much noise, the prediction will easily fail. Therefore, in experiments of this study, the data from two years operation is adopted to ensure that the data is capable to effectively interpret the operating status of the ORC. It also effectively exclude the missing values in the data pre-process. On the other hand, the highly correlated parameters to power generation could be defined through the correlation coefficient analysis. The adoption of the parameters can be ensured an effective established predictive model. If an ORC generator set which just start to be operated uses this method to predict power generation, this method will fail due to insufficient data.

## 5. Conclusions

Affected by the heat source temperature, pressure, and flow, thermal energy conversion is frequently unstable. Therefore, estimating the amount of power generated from thermal energy conversion is crucial for the effective operation of power systems. In this study, a deep learning neural network was proposed to estimate power generation from thermal energy conversion for the next 12 h.

The proposed method was based on a long short-term memory network, a type of recurrent neural network. ORC data from the steel industry were used to perform analysis. The results verified that the proposed method reduced the error by 24% compared with multilayer perceptron time series analysis. Regarding the use of a single or multiple variables to predict the amount of power generated, the LSTM models were more accurate compared with the ARIMA and MLP models in forecasting the next 12 h case, verifying that the proposed deep learning framework can be used to accurately predict the power generation of ORC systems.

In practice, power generation forecasting is conducive to the maintenance of power generators. When the prediction model is highly accurate, a logical tolerance interval can be employed to create a flexible warning threshold for generators and replace the conventional fixed threshold. Accordingly, abnormalities in generators can be detected in advance to perform early maintenance and reduce losses caused by tripping. The current research team will continue to improve the accuracy of power generation forecasting models. Different deep learning methods, such as sequence-to-sequence models and transformers, will be used to predict power generation. The optimization of these two methods will yield more accurate prediction results from time series data, facilitating the development of early diagnosis technology for power generators.

## Figures and Tables

**Table 1:** Statistics of power generated through thermal energy conversion

Mean (kW)	165.6
Standard deviation (kW)	28
Minimum value (kW)	100
Maximum value (kW)	246.8
Total number of data items	17,914

**Table 2:** Accuracy of the different models

Predicted Amount of Power Generated for the Next Hour	MAE	RMSE	MAPE
ARIMA(1,1,2)	0.5488	0.7498	453.5%
Univariate MLP	1.1772 (−8%)	1.4899 (−18%)	110.3%
Multivariate MLP	**1.2788**	**1.7932**	118.6%
Univariate LSTM	1.0605 (−17%)	1.3129 (−27%)	100.2%
Multivariate LSTM	1.1432 (−11%)	1.3130 (−27%)	100.0%
**Predicted amount of power generated for the next 12 h**	**MAE**	**RMSE**	**MAPE**
ARIMA(4,1,0)	9.1849	11.9719	285.1%
Univariate MLP	**1.1761**	**1.2295**	143.6%
Multivariate MLP	1.1573 (−2%)	1.1932 (−3%)	129.2%
Univariate LSTM	0.9457 (−20%)	1.1860 (−4%)	105.7%
Multivariate LSTM	0.8944 (−24%)	1.1396 (−8%)	100.0%
